# Global Change and Response of Coastal Dune Plants to the Combined Effects of Increased Sand Accretion (Burial) and Nutrient Availability

**DOI:** 10.1371/journal.pone.0047561

**Published:** 2012-10-15

**Authors:** Silvia Frosini, Claudio Lardicci, Elena Balestri

**Affiliations:** Department of Biology, University of Pisa, Pisa, Italy; Institut Mediterrani d’Estudis Avançats (CSIC/UIB), Spain

## Abstract

Coastal dune plants are subjected to natural multiple stresses and vulnerable to global change. Some changes associated with global change could interact in their effects on vegetation. As vegetation plays a fundamental role in building and stabilizing dune systems, effective coastal habitat management requires a better understanding of the combined effects of such changes on plant populations. A manipulative experiment was conducted along a Mediterranean dune system to examine the individual and combined effects of increased sediment accretion (burial) and nitrogen enrichment associated with predicted global change on the performance of young clones of *Sporobolus virginicus*, a widespread dune stabilizing species. Increased burial severity resulted in the production of taller but thinner shoots, while nutrient enrichment stimulated rhizome production. Nutrient enrichment increased total plant biomass up to moderate burial levels (50% of plant height), but it had not effect at the highest burial level (100% of plant height). The effects of such factors on total biomass, shoot biomass and branching were influenced by spatial variation in natural factors at the scale of hundreds of metres. These results indicate that the effects of burial and nutrient enrichment on plant performance were not independent. Their combined effects may not be predicted by knowing the individual effects, at least under the study conditions. Under global change scenarios, increased nutrient input could alleviate nutrient stress in *S. virginicus*, enhancing clonal expansion and productivity, but this benefit could be offset by increased sand accretion levels equal or exceeding 100% of plant height. Depletion of stored reserves for emerging from sand could increase plant vulnerability to other stresses in the long-term. The results emphasize the need to incorporate statistical designs for detecting non-independent effects of multiple changes and adequate spatial replication in future works to anticipate the impact of global change on dune ecosystem functioning.

## Introduction

Coastal sand dunes, along with the numerous valuable goods and services they provide [1]–[3], are threatened worldwide by both anthropogenic activities and climate change [4]–[8]. Physical and chemical changes associated with global change will potentially affect dune ecosystem structure and functioning in the coming centuries [7]–[12]. Dune plants play a fundamental role in determining the form, function and stability of dune systems [13]. Therefore, understanding and anticipating the response of individual plant species to abiotic changes is essential for developing effective coastal management and conservation strategies. However, this is problematic because in nature dune plants are subjected simultaneously to a variety of environmental stresses [14]–[19]. Although numerous studies have addressed dune plant adaptation to individual environmental stresses [20], [21], [17], [22], [23], [18], little is still known about how plants integrate the signals associated with concurrent stresses and adjust their growth accordingly. Climate-induced changes are expected to enhance the magnitude and frequency of existing natural and anthropogenic stress factors to levels that could exceed dune plant tolerance. Some changes could interact (synergically or antagonistically) in their effects, making it complex to predict the net effect on vegetation [24].

For example, alteration of global nutrient cycles due to the use of fertilizers and increased atmospheric deposition will enhance the inputs of nutrients, particularly nitrogen (N) and phosphorus (P) [25]–[27]. Predictions indicate that by 2050, nitrogen deposition may double, with some regions of the world reaching 50 kg N ha^−1^ yr^−1^ [28], [25]. The availability of N in soils plays a fundamental role in influencing plant community composition and stability [29]. Increased N deposition will likely be one of the greater drivers of plant biodiversity loss at the global scale over the coming century [24], [25]. Coastal sand dunes are nutrient poor habitats and plant productivity has been found to be limited by both N and P [30], [16]. Studies have shown that increased nutrient inputs would favour the growth of some species (tall grass species, in particular) while would make others more vulnerable to disturbances and stresses [30]–[33], [23]. However, it is unclear whether increased atmospheric N deposition alone can induce encroachment by dune grasses or whether other factors are involved [30], [23]. On the other hand, changes in atmospheric circulation will increase the frequency of extreme wind events, leading to more frequent episodes of sand accretion in some areas [34]–[36]. Burial by wind-deposited sand is one of the major physical stresses that can alter dune community composition, distribution and abundance [14], [37], [15], [16]. There is large variation in the degree of adaptation to burial among dune species. Substantial variation also occurs within a species in function of life history stage, season and burial severity, in terms of depth relative to the height of plants [14]–[16] and frequency. Many plant species are able to emerge from low or moderate levels of burial (less than 50% of plant height) by elongating shoots (positive growth response), but only few species (i.e., *Ammophila* spp.) are able to withstand deeper burial [14]–[16], [38]. Other species are unaffected (neutral growth response) or inhibited (negative growth response) by burial [14]–[16], [37], [38]. However, recurrent frequent shallow burial events can be more damaging than a single event of greater magnitude [39], and juveniles may be especially sensitive to such events [40]. Therefore, species intolerant to recurrent shallow burial events are expected to be prevented from occupying mobile dunes and spatially replaced by more tolerant species under wind pattern change scenarios. However, there is still no general consensus on the physiological mechanisms behind burial growth response [38]. Potential mechanisms include shifts in resource (such as biomass and nutrients) allocation from below-ground to above-ground components, remobilization of stored resources, changes in photosynthetic rate or other attributes, and reduction of the dry mass cost of producing new leaves and elongating stems [14]–[16], [41]. More recent studies have shown that the ability of mobile dune species to respond to burial may largely depend upon the availability of nutrients [20], [22]. However, severe sand deposition episodes may drastically modify physical or chemical micro-environment characteristics and also favour the activity of anaerobic microorganisms [14], [30], potentially reducing or preventing plant nutrient uptake. The possible interaction between nutrient availability and burial implies that their net effect on a given plant species may be larger than, or smaller, than the expected individual effects. To our knowledge, very few studies were designed to test in the field the potential non-independent effects of more than one factor on coastal dune vegetation in relation to global change [23].

In this study we investigated the response of young clones of *Sporobolus virginicus* Kunth, a pioneer dune herbaceous species [42]–[43], to the individual and combined effects of increased repeated burial levels and N enrichment to gain more insights into how mobile dune plants will change in the future. *Sporobolus virginicus* was chosen as model because of its worldwide distribution and fundamental role in stabilizing mobile dune substrates [43]–[45]. The burial tolerance limit of this species has not been investigated yet. We hypothesized that the effects of the two investigated factors on plant performance would be non-additive (interacting). Specifically, we expected that plant growth might be nutrient limited, and enhanced nutrient availability might stimulate plant productivity alleviating physiological stress under increased burial conditions. As an alternative, increased nutrient input might have no positive effect on plant growth under increased burial conditions because of alterations of sediment properties or metabolic shift from an energy-producing to an energy-consuming state [16]. Since in coastal habitats a number of factors, including topographic features, edaphic conditions and resource distribution, can vary at the scale of microhabitat [8], [46]–[49], the effects of the experimental factors were tested across different spatial scales, from tens to hundreds of meters, along a Mediterranean dune system. To our knowledge, no previous field studies have explicitly assessed whether the response of a species to abiotic factors was consistent in space along its distribution zone (horizontally) within a dune system.

## Materials and Methods

### Ethics Approval

All necessary permits were obtained for the described field studies. The study site belongs to Rosignano Marittimo Municipality which issued the permission for all our field studies. The species is not protected in Italy, but the habitat has a priority status in Europe (Habitat Directive 92/43/EEC). In field studies we tried our best not to damage seedlings or individuals.

**Figure 1:**
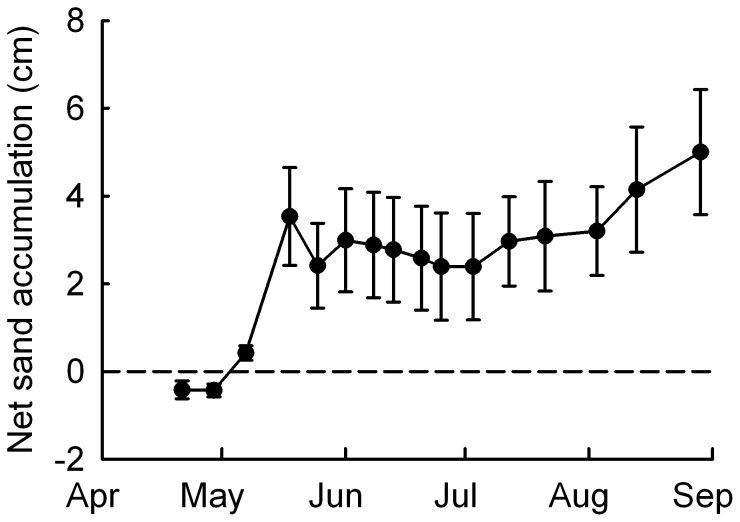
Ambient burial levels in the study area. Net changes in sand accumulation (cm) relative to erosion pins (n = 24) placed along a transect parallel to the coastline at the study dune system. Data were recorded weekly from 13 April to 29 August 2010. Bars represent ± SE.

### Study Species

*Sporobolus virginicus* (Poaceae) is a perennial, herbaceous clonal species widely distributed along tropical and subtropical lagoon, sand beaches and estuaries [42]. The tolerance of this species to waterlogging and salinity can account for exploitation of a wide range of coastal environments [43], [50]. In Italy, the presence of this species has been reported only recently because it was retained as a distinct species and referred to as *Sporobolus pungens* Schreber Kunth [51]. Clones form horizontal rhizomes that produce branched or solitary ascending culms and adventitious roots at each node. Rhizome connections and roots form large networks which efficiently stabilize substrates and initiate the recovery of mobile dunes following disturbance [42]. In the Mediterranean dunes, the vegetative growth of this species occurs from late winter (February) to the end of autumn, and the reproductive season generally lasts from May to September [52]. Flowers consist of spike-like hermaphroditic panicles, 2–10 cm long. Despite *S. virginicus* can produce seeds [53], recruitment from seed is considered rare in nature [54].

**Figure 2:**
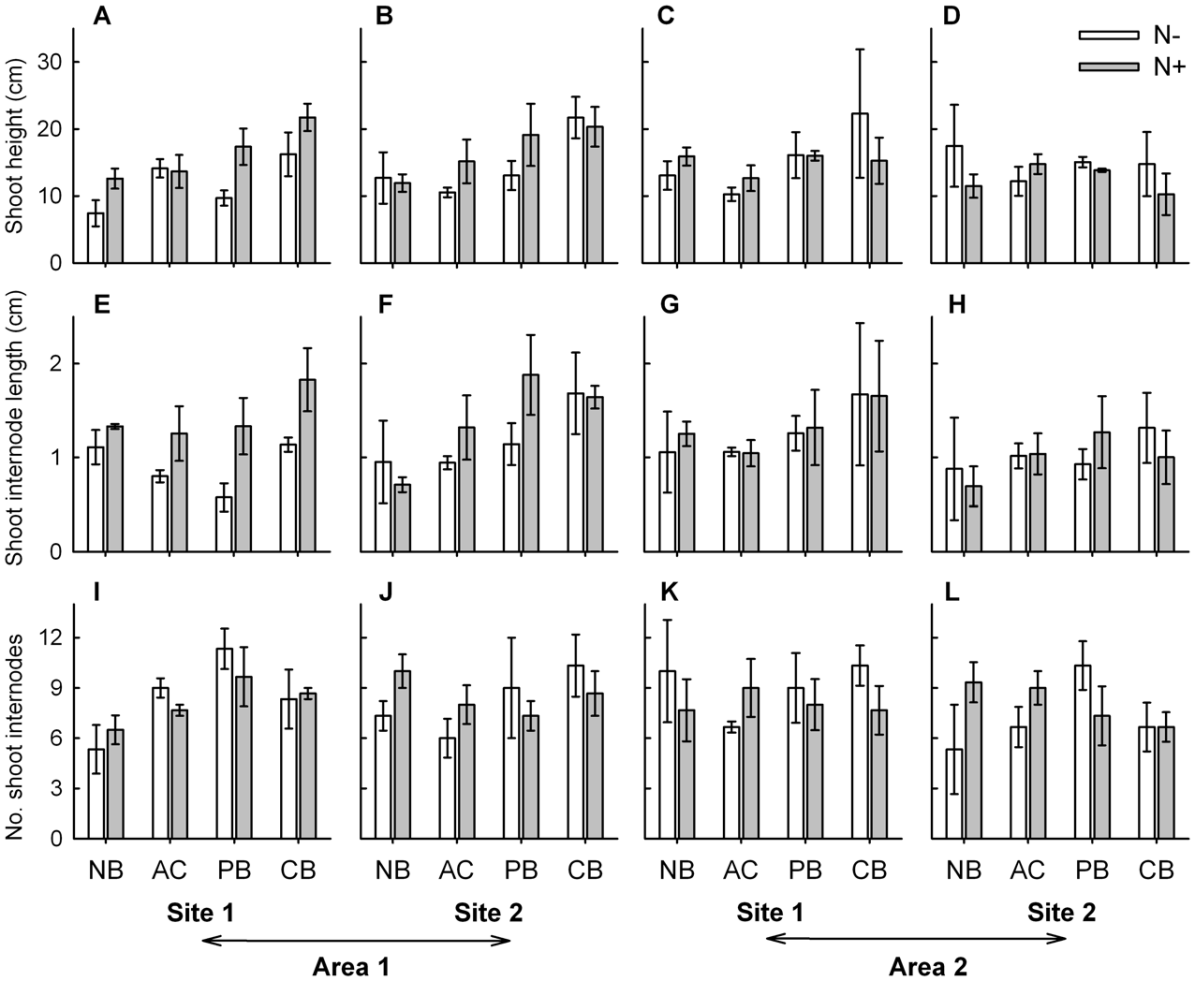
Morphological variables of *Sporobolus virginicus* clones subjected to different experimental treatments. Mean (±1 SE) maximum shoot length (A, B, C, D), shoot internode length (E, F, G, H) and number of shoot internodes (I, J, K, L) of plants grown in the two sites within each of the two areas. N−  =  no nutrient added, N+  =  nutrient addition; NB  =  no burial, AC  =  artifact control, PB  =  partial burial, CB  =  complete burial. n = 3.

**Figure 3:**
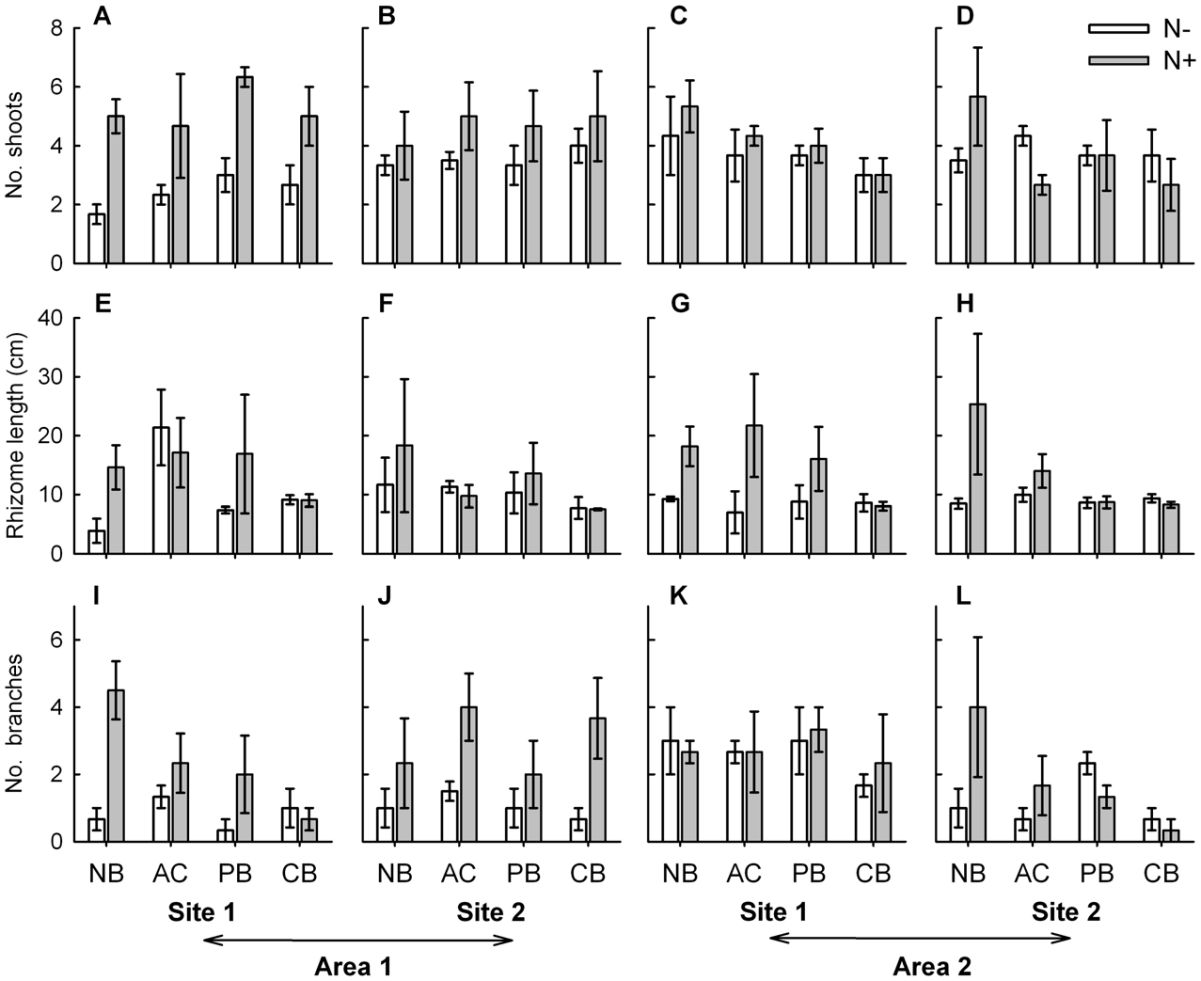
Morphological variables of *Sporobolus virginicus* clones subjected to different experimental treatments . Mean (±1 SE) number of shoots (A, B, C, D), rhizome length (E, F, G, H) and number of branches (I, J, K, L) of plants grown in the two sites within each of the two areas. N−  =  no nutrient added, N+  =  nutrient addition; NB  =  no burial, AC  =  artifact control, PB  =  partial burial, CB  =  complete burial. n = 3.

**Figure 4:**
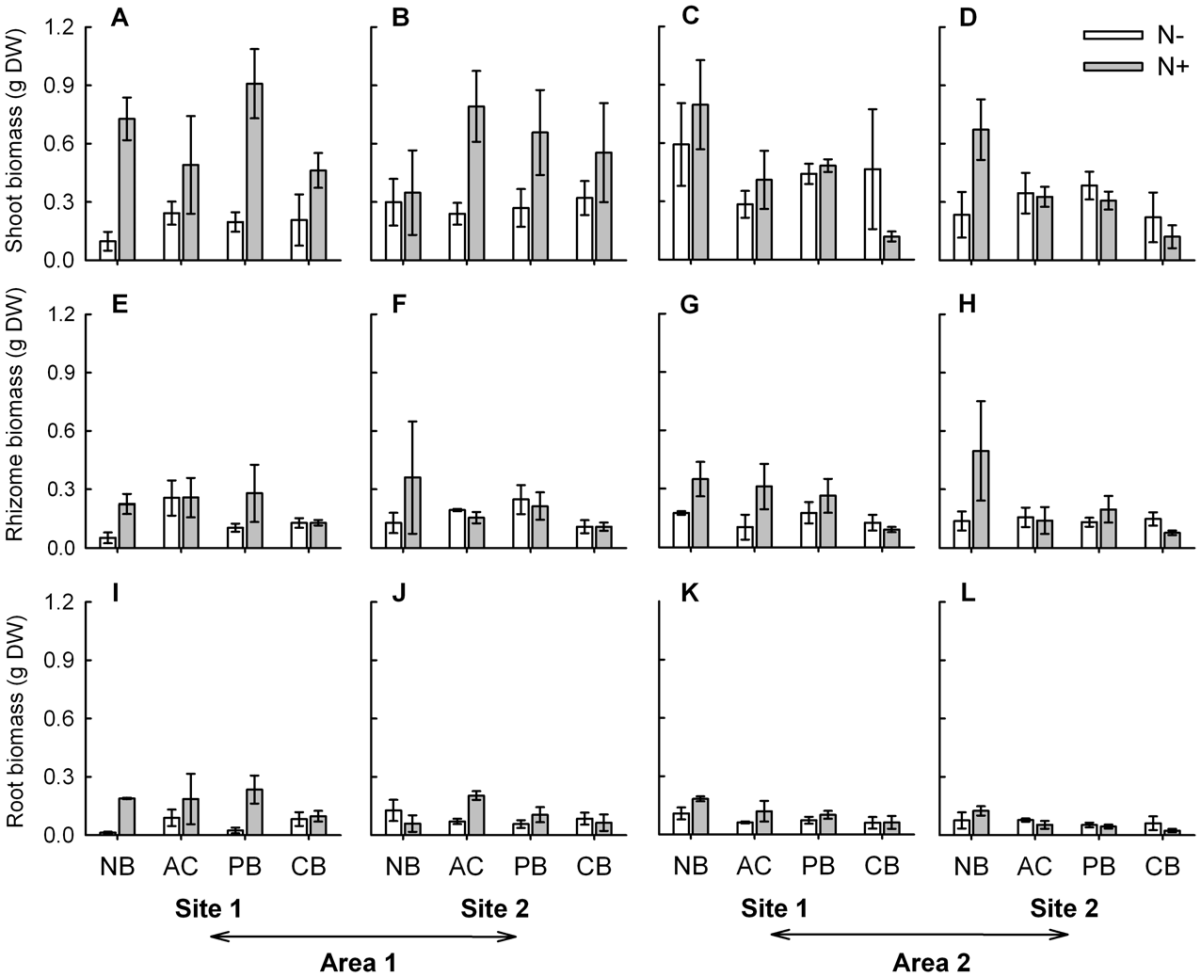
Biomass of main plant parts of *Sporobolus virginicus* clones subjected to different experimental treatments. Mean (±1 SE) biomass of shoots (A, B, C, D), rhizome (E, F, G, H) and roots (I, J, K, L) of plants grown in the two sites within each of the two areas. N−  =  no nutrient added, N+  =  nutrient addition; NB  =  no burial, AC  =  artifact control, PB  =  partial burial, CB  =  complete burial. n = 3.

### Study Locality and Plant Material Preparation

The study was conducted on the coastline of Rosignano Solvay in the north-western Italy (43°22′43.10′′N, 10°26′15.77′′E). The coastline is characterized by a mobile dune system (2.5 km long) that runs parallel to the shoreline; the height of dunes varies from 1.0 to 8.5 m from the 0 m water level. Winds are predominantly from the south- and south-west during the winter and from northwest in the summer, favouring a net inshore movement of sand. Available data indicated that the frequency of extreme winds in summer considerably increased during the past decades [55]. The climate is typical Mediterranean: the mean daily temperature of the coldest month (January) is 5°C, while that of the warmest month (July) is 25°C (data from local meteorological stations). The substrate of beach and mobile dunes is fine strongly alkaline sand (98.7%, silt 0.4%; clay 0.9%; pH >8.3), rich in calcium carbonate (CaCO**_3,_** 86.59%) [56]. Dune system is dominated by *Ammophila arenaria* (L.) Link (European beachgrass) and *Elymus farctus* (Viv.) Runemark ex Melderis. *S. virginicus* is abundant on the upper beach and first mobile dune ridge where it forms large monospecific patches parallel to the shoreline. The net sand accretion rate at the study dune system over the study period was estimated by monitoring changes in the level of sand deposition relative to erosion pins (24 pins) randomly placed along a transect parallel to the shoreline. The height of pins was weekly measured with an accuracy of 1–2 mm.

Because seeds of *S. virginicus* are rare [53] and defining individual clones in nature is difficult, prior to the experiment (October 2009) clones were produced in a nursery by vegetative propagation from rhizome fragments (with two-nodes and about 8 cm in length) collected at the edge of established patches at the study site. These fragments were rooted into pots (30 cm depth and 10 cm diameter) containing a 1∶1 (v/v) mixture of beach sand and potting compost, and maintained outdoor. In April 2010, rooted plants were extracted from sediment for morphological measurements (rhizome length, number of shoots, number of branches and maximum shoot height). This material was representative of plants established in nature from clonal fragments generated during autumn-winter storms. Understanding the ability of such plants to withstand incoming environmental changes is critical because of their major role in expanding populations and recovery after disturbance [57], [58]. To remove the possible effect of different clone size, similar-sized plants (96 plants) were selected and transported to the study system. Two areas, separated by hundreds of meters, were randomly chosen along the dune system and within each area two sites, ten of meters apart, placed on the first dune ridge were selected at random. The sites (2–3 m wide dune stretches) had an elevation of 1.2–1.5 m from the 0 m water level, and their distance from the shoreline was ca. 80 m. Plants (24 plants) were individually transplanted in random positions on zones of bare substrate within a natural *S. virginicus* population within each site. Plants were separated from each other by at least 0.5 m to avoid possible contamination between treatments. Three plants, dead soon after planting, were substituted with plants maintained in the nursery as reserve. The study dune tract was fenced in attempt to prevent anthropogenic interference.

**Table 1:** ANOVAs for the effects of area, site, burial height, nutrient availability and their interactions on morphological and growth variables of *Sporobolus virginicus* clones.

		Shoot height (cm)		Shoot internode length (cm)		No. shoot internodes	
Source	d.f.	MS	*F*	MS	*F*	MS	*F*
Area = A	1	0.6	0.45	0.13	0.25	1.15	0.25
Burial = B	3	2.99	4.50**	1.16	4.24**	9.21	2.03
Nutrient = N	1	1.4	0.7	0.87	1.12	0.02	0
Site(A) = S(A)	2	1.32	2.31	0.52	1.9	4.6	0.66
A × B	3	0.30^a^		0.02^a^		4.54^b^	
A × N	1	2.01	3.53	0.77	2.82	0.00^a^	
N × B	3	0.42	0.75	0.26	0.95	16.05	2.31
B × S(A)	6	0.32^a^		0.29^a^		10.26^b^	1.53
N × S(A)	2	0.21^a^		0.17^a^		5.35^a^	
A × N × B	3	0.36^a^		0.1^a^		2.87^a^	
N × B × S(A)	6	0.18^a^		0.07^a^		6.8^b^	
Residual	62	0.67^a^		0.32^a^		7.33^a^	
SNK		CB >PB = NB = AC		CB >PB > NB = AC			
		**No. shoots**		**Rhizome length (cm)**		**No. branches**	
**Source**	**d.f.**	**MS**	***F***	**MS**	***F***	**MS**	***F***
Area = A	1	1.15	1.04	0.02	0.36	0.18	0.2
Burial = B	3	0.8	0.18	2.06	2.34	0.38	1.2
Nutrient = N	1	32.09	1.97	8.14	9.24**	2.01	2.1
Site(A) = S(A)	2	1.11	0.46	0.07	0.08	0.89	6**
A × B	3	4.36	1.94	0.73^a^	0.32	2.13	
A × N	1	16.25	3.58	0.91^a^		0.96	6.41*
N × B	3	2.79	1.25	2.02	2.29	0.18	0.88
B × S(A)	6	0.91^a^		0.92^a^		0.13^a^	
N × S(A)	2	4.54	2.03	0.56^a^		0.05^a^	
A × N × B	3	2.21^a^		1.09^a^		0.01^b^	
N × B ×S(A)	6	1.68^a^		0.49^a^		0.3^b^	
Residual	62	2.43^a^		0.92^a^		0.15^a^	
SNK						A2: S1> S2; A1: N+ > N-	
		**Shoot biomass (g DW)**		**Rhizome biomass (g DW)**		**§ Root biomass (g DW)**	
**Source**	**d.f.**	**MS**	***F***	**MS**	***F***	**MS**	***F***
Area = A	1	0.03	0.37	0	1	0.02	1.9
Burial = B	3	0.13	0.81	0.07	3.00*	0.01	1.24
Nutrient = N	1	1.04	1.42	0.15	6.88*	0.05	0.89
Site(A) = S(A)	2	0.09	1.45	0	0.08	0.01	1.92
A × B	3	0.16	2.62	0.02^a^		0.01	1.85
A × N	1	0.73	11.56**	0.01^a^		0.02^c^	
N × B	3	0.12	1.82	0.07	3.29*	0.01	1.43
B × S(A)	6	0.04^a^		0.01^a^		0.00^a^	
N × S(A)	2	0.04^a^		0.00^a^		0.02^c^	
A × N × B	3	0.09^a^		0.01^a^		0.01^b^	
N × B × S(A)	6	0.07^a^		0.02^a^		0.1^b^	
Residual	62	0.07^a^		0.02^a^		0.00^a^	
SNK		A1: N+ > N-		NB & AC: N+ > N-			

a, b, c Denote post-hoc pooling, *P*>0.25; new *F*-values are given for those tested against the pooled term. Results of SNK tests are also reported. NB  =  no burial, AC  =  artifact control, CB  =  complete burial, PB  =  partial burial. A1, A2 =  area 1 or 2, S1, S2 =  site 1 or 2, N−  =  no nutrient added, N+  =  nutrient added. *  =  *P*<0.05, **  =  *P*<0.01. §  =  variances were heterogeneous (Cochran’s *C* test, *P*<0.05) and α = 0.01 was adopted.

**Figure 5:**
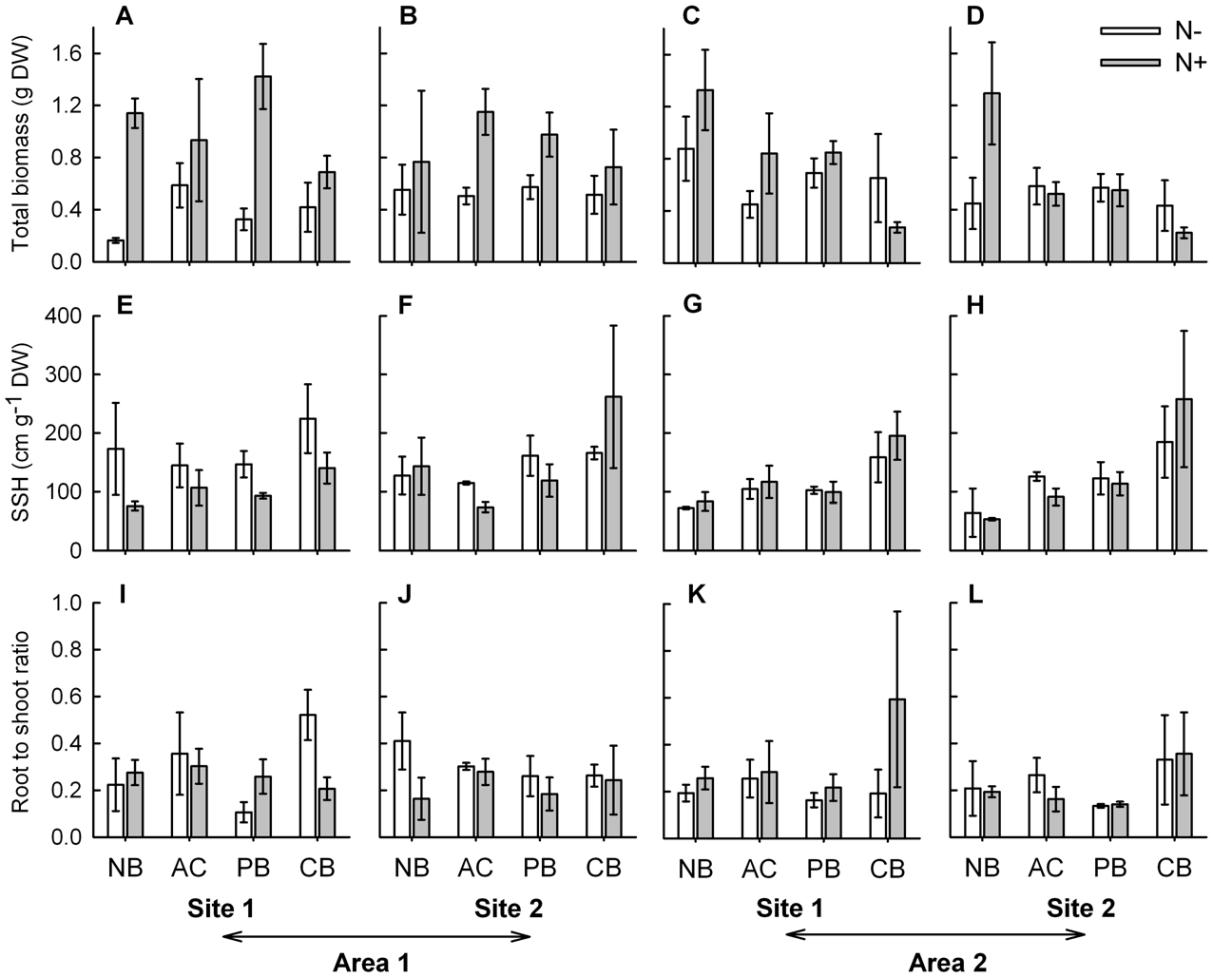
Total biomass, specific shoot height and allocation of *Sporobolus virginicus* clones subjected to different treatments. Mean (±1 SE) total biomass (A, B, C, D), specific shoot height, SSH (E, F, G, H), and root to shoot ratio (I, J, K, L) of plants grown in the two sites within each of the two areas. N−  =  no nutrient added, N+  =  nutrient addition; NB  =  no burial, AC  =  artifact control, PB  =  partial burial, CB  =  complete burial. n = 3.

### Experimental Design

After acclimation (late May 2010) to local environmental conditions, plants growing in each site were randomly assigned to one of six treatments: moderate sand burial resulting in the cover of 50% of shoot height (partial burial, PB/N-), high sand burial resulting in 100% cover of shoot height (complete burial, CB/N-), no sand burial and nutrient enrichment (NB/N+), partial sand burial and nutrient enrichment (PB/N+), complete sand burial and nutrient enrichment (CB/N+), no sand burial and no nutrient enrichment (ambient conditions, NB/N-). Before the start of the experiment, rhizome length, number of shoots, number of branches and maximum shoot height of plants were measured to test whether the plants assigned to different treatments did not differ significantly from each other in morphology and size. A preliminary study has shown that the mean annual net sand accretion recorded at the study site corresponded to a burial of 50% of shoot height (about 5 cm; personal observation). Woody frames (30×30 cm in size) were placed around each plant and filled with sand to maintain the assigned burial level. To elevate nitrogen concentration and reduce leaching, a single dose of a commercially available fertilizer (COMPO, K+S Agricoltura Spa, g/g ratio N-P-K 13-6-9) formulated for a 3-month complete element release was inserted into the sediment. Treatment N is equal to a dose of 14 kg N ha^−1^, and treatment P to 6.6 Kg P ha^−1^. The N dose is equivalent to the deposition estimates (15–20 kg N ha^−1^ year^−1^) predicted for the 2050 in the Mediterranean basin [28], [25]. In addition, two treatments, in which empty frames were placed around plants were used to control for possible artifact effect: artifact control and no nutrient enrichment (AC/N-) and artefact and nutrient enrichment (AC/N+). There were three replicates for each treatment.

**Table 2:** ANOVAs for the effects of area, site, burial height, nutrient availability and their interactions on total biomass, specific shoot height (SSH) and root to shoot ratio of *Sporobolus virginicus* clones.

		Total biomass (g DW)		SSH (cm g^−1^ DW)		Root to shoot ratio	
Source	d.f.	MS	*F*	MS	*F*	MS	*F*
Area = A	1	0.02	0.14	9936	10.01	0.05	2.63
Burial = B	3	0.39	0.71	49224	9.66***	0.12	2.44
Nutrient = N	1	2.82	3.08	2692	0.53	0	0.04
Site(A) = S(A)	2	0.18	1.34	993	0.17	0.02	0.35
A × B	3	0.55	4.16**	5058^a^		0.01^a^	
A ×N	1	0.92	6.92*	9523^a^		0.11^a^	
N × B	3	0.48	3.62**	4553	0.89	0.01	0.23
B × S(A)	6	0.03^a^		2244^a^		0.01^a^	
N × S(A)	2	0.11^a^		8647	1.7	0.06^a^	
A × N × B	3	0.12^a^		115^a^		0.06^a^	
N × B × S(A)	6	0.14^a^		3287^a^		0.05^a^	
Residual	62	0.14^a^		5717^a^		0.05^a^	
SNK		A2: NB = AC >PB > CB;		CB >PB > NB = AC			
		NB: A2> A1;					
		N-: A2> A1; N+: A1> A2;					
		A1 & A2: N+ > N-					
		N+: NB = AC = PB > CB;					
		NB & AC & PB, N+> N-					

a Denotes post-hoc pooling, *P*>0.25; new *F*-values are given for those tested against the pooled term. Results of SNK tests are also reported. NB  =  no burial, AC  =  artifact control, CB  =  complete burial, PB  =  partial burial. A1, A2 =  area 1 or 2, S1, S2 =  site 1 or 2, N−  =  no nutrient added, N+  =  nutrient added.

* = *P*<0.05.

** = *P*<0.01.

*** =

*P*<0.001.

Plants were monitored at weekly intervals until the end of the experiment (August 2010). At each census, plants were carefully inspected for herbivore damage because of its potential influence on plant growth [59], and the height of newly produced shoot tissue was measured. As the experiment was trying to simulate recurrent burial summer events, plants were reburied weekly to the experimental originally attributed burial level. The sand used for burial treatments during the course of the experiment was collected to a depth of maximum 10 cm closely to the treated plants; it was sieved to remove propagules and extraneous material prior to the use.

At the end of the experiment, the number of plants that had recovered from burial was recorded. The root system of surviving plants was gently excavated to remove plants from the substrate. Plants were transported to the laboratory where morphological characteristics that were expected to be affected by the investigated factors on the basis of available literature (horizontal rhizome length, number of vertical shoots, maximum shoot height, number of branches, number and length of vertical internodes measured on the highest shoot, and the number of reproductive shoots per plant) [20], [14]–[16], [22], [41] were recorded. Plants were then separated into shoots, roots and rhizomes and dried at 60°C until they reached constant weight (dry weight, DW) to determine the respective biomasses. In addition, to investigate plant response in terms of resource allocation and efficiency of production, root to shoot ratio and specific shoot height (SSH) were calculated. Root to shoot ratio, which reflects the differential investment of photosynthates between the above-ground and below-ground organs [60], was calculated by dividing root by shoot biomass (g g^−1^ DW). Specific shoot height, which is considered an indicator of the dry mass cost of producing shoots [41], was calculated as the ratio between total shoot height and shoot dry weight (cm g^−1 ^DW).

### Data Analysis

Data on morphological and growth variables were analysed separately for the time period before the treatments were initiated (May 2010) and for the time period following the treatments (August 2010). Initial data were analysed throughout multivariate analysis of variance by permutation, PERMANOVA [61], according to a randomized ANOVA design that included the orthogonal factors area (two levels, random) and treatment assignation (eight levels, random), and the factor site (two levels, random) nested within area and orthogonal to treatment assignation. Final data were analysed using PERMANOVA according to mixed model ANOVA design that included the orthogonal factors area (two levels, random), burial (four levels, fixed) and nutrient (two levels, fixed), and the factor site (two levels, random) nested within area and orthogonal to burial and nutrient. Because of the loss of two plants in one area, unbalanced PERMANOVA with type III sums of squares was performed [62]. Since significant effects were detected in PERMANOVA, separate ANOVAs were performed for all investigated variables according to the same model. Separate ANOVAs were also conducted on total plant biomass, root to shoot ratio and specific shoot height. Missing replicates were substituted with the mean of that particular combination of treatments and two degrees of freedom were subtracted from the total degrees of freedom of the residual mean square [63].

Prior to PERMANOVA, data were normalized and dissimilarities calculated as Euclidean distances. Significance levels were calculated from 9999 permutations of the residuals under the reduced model. Whenever possible, *post hoc* pooling of mixed terms of the model was performed to increase analysis power [64]. When a significant effect was found, *post hoc* pair-wise comparisons (PERMANOVA *t* statistic and 999 permutations) were used to distinguish between means. For some terms, there were not enough permutable units to get a reasonable test by permutation, so *P*-values were obtained using a Monte Carlo random sample from the asymptotic permutation distribution [62]. Statistically significant terms were checked for differences in multivariate group dispersion with the permutational analysis of multivariate dispersions (PERMDISP) [65]; pair-wise comparisons of multivariate dispersion were also performed between all couples of groups. Prior to performing ANOVAs, data were tested for normality and homoscedasticy, and transformed if necessary. Whenever data transformation failed to achieve homogeneity of variances, the analysis was performed on untransformed data with α = 0.01 [64]. When significant effects were detected, means were compared through the Student-Newman-Keuls (SNK) test [64]. As for the multivariate analysis, *post hoc* pooling of mixed interaction term was applied whenever possible.

PERMANOVA and PERMDISP were run through PRIMER v6 (Primer-E Ltd., Plymouth) [66] with PERMANOVA add-on software, while statistical software R version 2.12.2 [67] and R package “GAD” [68] were used for ANOVAs.

## Results

Before applying the treatments, the plants randomly assigned to different treatments were of equal age (six months) and similar size (see Table S1). They had on average 1.5 (±0.1 SE) shoots and 2.7 (±0.1) branches; shoot height was on average 10 (±0.2) cm and the horizontal rhizome was 8.7 (±0.2) cm long. Two of the 96 transplanted individuals disappeared during the study period because of unknown factors. At the end the experiment, survived plants had produced at least one new shoot each. No inflorescences were observed during the study period and no sign of herbivore damage was detected in plants. All plants exposed to increased burial were emerged above the sand surface. The mean height of sand deposed on plants exposed to partial burial over the course of the experiment was 8 cm (±0.4) while the height of sand deposed on plants exposed to complete burial was 19.2 cm (±1.6). These values were higher compared to the sand deposition level experienced by plants grown at ambient conditions over the study period along the dune system (Fig. 1). No traces of fertilizer were detected in the soil at plant harvesting, indicating that the release of nutrients was complete in the experimental period.

A significant interaction between nutrient availability and burial on whole plant response was detected (Table S2). Overall, plants grown under nutrient enhanced conditions differed from those grown under nutrient ambient conditions, and partially buried plants differed from completely buried plants but only when grown under nutrient enhanced conditions (Table S3). No difference in multivariate dispersion among significantly differing groups was detected (pair-wise PERMDISP test: unfertilized vs. fertilized for unburied plants, *t* = 0.99, *P* = 0.361; fertilized and completely buried vs. fertilized and partially buried plants, *t* = 1.08, *P* = 0.373), indicating that the effects reported above were effectively due to investigated factors, and not to a different multivariate dispersion among groups. A significant interaction between nutrient supply and area was also detected (fertilized plants differed from unfertilized ones only in one of the two areas, Table S3), but this could be due to different multivariate dispersion of the groups (PERMDISP test for unfertilized vs. fertilized plants in area 1, *t* = 3.46, *P* = 0.006) rather than to the investigated factors.

The results from separate ANOVAs showed that for three out of the nine investigated morphological and growth characteristics, the effect of burial and nutrient availability was additive (i.e., no significant interaction occurred), while for one variable it was non-additive (significant interaction occurred). Burial alone significantly affected shoot height and shoot internodes length (Table 1). Plants grown under completely buried conditions had on average taller shoots (ca. 35–40%) than those grown under ambient burial conditions, while those grown under partially buried conditions had an intermediate height (Fig. 2A–D). Shoot internodes of completely buried plants were significantly longer (about. 20%) than those of partially buried plants that in turn were about 15–20% longer than those at ambient burial conditions (Table 1, Fig. 2E–H). Instead, nutrient availability alone significantly affected rhizome length (Table 1). The rhizome of plants grown under enhanced nutrient conditions was about 30% longer compared to that of plants grown under ambient nutrient conditions (Fig. 3E–H). Burial and nutrient availability in combination affected rhizome biomass. Nutrient addition resulted in a threefold increase in rhizome biomass but only under ambient burial conditions (Fig. 4E–H; Table 1). Results also indicated a significant interaction between nutrient supply and area for two variables, shoot biomass and number of branches. Both shoot biomass and number of branches increased under enhanced nutrient conditions, but only in one of the two areas (area 1, Figs. 3I–L and 4A–D; Table 1). For this latter variable, a significant effect of site was also observed (Fig. 4A–D; Table 1). Finally, for the remaining three variables, number of vertical internodes, number of shoots and root biomass, no significant effect of the investigated factors, alone or in combination, was observed (Figs. 2I–L, 3A–D and 4I–L; Table 1).

Total plant biomass was significantly affected by the interaction between burial and nutrient availability (Table 2). When grown under enhanced nutrient conditions, the biomass of plants increased significantly as compared to that of plants grown under ambient nutrient conditions, except when plants were completely buried. Under enhanced nutrient conditions, the total biomass of completely buried plants was about half of that of plants partially buried or grown at ambient burial conditions (Fig. 5A–D). Significant interactions between area and burial, and between area and nutrient supply, were also detected (Table 2). The biomass produced by plants grown at ambient burial conditions was greater than that of plants subjected to partial burial, and the biomass of these latter was in turn greater than that of plants grown under complete burial but only in one of the two areas (area 2). Significant differences in the biomass of plants grown at ambient burial conditions between areas were also found. In both the areas, the biomass of fertilized plants was greater than that of unfertilized ones. The biomass of unfertilized plants was higher in the area 2 than in area 1, while the opposite pattern was found for fertilized plants (Fig. 5A–D). Specific shoot height was significantly influenced by burial alone, and increased with the increase of the severity of burial, from 99.23 (±14.01) cm g^−1^ DW at ambient conditions to 198.6 (±22.62) cm g^−1^ DW under completely buried conditions, (Fig. 5E–H; Table 2). Finally, the root to shoot ratio was much less than 1 (ranging from 0.22±0.003 to 0.28±0.004 g g^−1^ DW at the four sites) indicating that a substantial larger portion of biomass was concentrated above-ground (Fig. 5I–L). No significant differences were detected for this variable among treatments, indicating that individuals did not change their biomass allocation pattern in response to any of the investigated factors (Table 2).

## Discussion

Burial by sand has been assumed to be a major environmental stress reducing plant performance in dune species, especially in their early life history stages [14]–[16]. Our results showed that young clones of *S. virginicus* were able to recover from increased sand accretion levels that corresponded to about four times the mean maximum burial depth they naturally experienced over the study period, by elongating internodes in vertical shoots regardless of nutrient availability. This indicates that this species possesses an inherent ability to respond to burial that is consistent with its role of primary colonizer of dune areas of high sand movement [42]. Contrasting results emerged from previous studies on the mechanisms underlying compensatory shoot growth in response to burial in dune plants [20], [22], [41], [38]. A number of studies reported evidence of shifts in resources from below-to above ground plant parts [40], [69], while other studies failed to detect it [70] or indicated that shifts were only possible at low or moderate burial levels (up to 66% of plant height) [38]. In the current study, stimulation of shoot elongation by burial might not be attributed to an increased biomass investment in the above-ground structures, as the root to shoot ratio was unaffected by burial. Instead, the increased specific shoot height indicates that the resources required for emerging from sand were obtained by reducing shoot production costs (i.e., more shoot length was produced with the same amount of biomass) and remobilizing resources from buried shoot tissue. Such response, which has been rarely observed in dune plants [41], could be adaptive, as it minimises nutrient use but maximises shoot growth, enabling the species to survive on mobile substrate under nutrient-limited conditions. However, depletion of stored reserves and production of thinner shoots due prolonged burial events may increase the vulnerability of plants to other abiotic stresses.

Previous studies demonstrated that nutrient inputs from atmospheric deposition favour the growth of graminoids and nitrophilous species, resulting in perturbation of competitive hierarchy among dune plant species with consequent loss of diversity and conservation habitat value [30]–[32], [23]. Here, nutrient addition alone resulted in increased rhizome production in *S. virginicus*, confirming our hypothesis that the species growth was nutrient limited under ambient conditions. Similarly to burial, increased nutrient availability did not induce shifts in biomass allocation from below- to above-ground components. This is not in agreement to that previously observed in most dune species [32], [71], [37]. Thus, *S. virginicus* can be considered as “form-conservative”, i.e., the form and the allocation of biomass of a plant of given size is the same irrespective of the nutrient microenvironment [72].

According to our prediction, the effects of nutrient enrichment and increased burial on total biomass production were non-independent. However, while total biomass increased in response to nutrient enrichment under partially buried conditions and at ambient conditions, no consistent biomass increase was found under completely burial conditions. Consequently, increased nutrient availability was ineffective in ameliorating plant stress under complete burial. This suggests that burial-driven alterations in the sediment micro-environment reduced the availability of nutrients or the uptake efficiency in plants [14], [30]. Another explanation might be the shift observed in completely buried plants from an energy-producing to an energy-consuming state [16].

Finally, the variability in the response of *S. virginicus* to nutrient availability and burial observed at small spatial scales (hundreds of metres) along the study dune system for some growth and architecture variables indicates that local natural factors, such as dune topography and light intensity [8], [46]–[49], might have interacted with the manipulated factors. Although a number of studies indicated that abiotic factors may vary at small spatial scales, not only across (transversally) but also along the shore (horizontally) on a dune system [46]–[48], the majority of the previous experimental studies on dune plant response to abiotic factors lacked of spatial replication, making it difficult to generalize their results.

In conclusion, the present study suggests that increased nutrient availability and burial severity may interact in their effects on dune plant performance, thus their combined effects may be not predicted by knowing the individual effects. According to recent models [28], [25], current N atmospheric deposition rate at the study site was about 5 kg N ha^−1^ y^−1^. Total N input (current plus experimentally imposed) was about 15.5 kg ha^−1^ in three months, a value close to the annual deposition level predicted for the 2050 in the Mediterranean basin [28], [25], and also within the range of the critical loads (10–20 kg ha^−1^ yr^−1^) for European foredune systems [23], [30]–[33]. We therefore expect the changes in atmospheric N deposition in the coming decades could alleviate nutrient stress in newly regenerated clones of *S. virginicus* enabling them to produce longer rhizomes and exploit a larger number of nutrient-rich patches or pulses on mobile dunes, resulting in higher plant cover. However, in areas with sand accretion levels equal or exceeding plant height the benefits of increasing nutrient input could be offset by burial stress, and prolonged burial exposure could reduce plant performance. Further studies on the effects of multiple factors on different species that could potentially outcompete *S. virginicus* on mobile dunes are needed to improve predictions about the possible consequences for population expansion in the long-term**.** The results also emphasize the need to incorporate statistical designs for detecting interactions between stressors, non-independent effects of multiple stresses and adequate spatial replication in future works. A better understanding of how dune plants will respond to the cumulative effects of abiotic changes is critical to establish effective conservation approaches and restoration actions in order to mitigate the effects of incoming global change on coastal dune structure and functioning.

## Supporting Information

Table S1 **PERMANOVA on Euclidean distances of plants assigned to different treatments replicated in two sites within two areas selected at random along the study dune system at the beginning of the experiment (May 2010).**
(DOC)

Table S2 **PERMANOVA on Euclidean distances of plants subjected to different treatments replicated in two sites within two areas selected at random along the study dune system at the end of the experiment (August 2010).**
(DOC)

Table S3 **Results of** ***a posteriori*** **pair-wise comparisons for the significant interaction terms, burial × nutrient and area × nutrient, detected by PERMANOVA (Table S2).**
(DOC)
