# Supplementary material for: Global Change and Response of Coastal Dune Plants to the Combined Effects of Increased Sand Accretion (Burial) and Nutrient Availability
Source: PLoS One. 2012 Oct 15;7(10):e47561. doi: 10.1371/journal.pone.0047561 (PMC3471884; doi:10.1371/journal.pone.0047561)
Supplement: Table S1 — PERMANOVA on Euclidean distances of plants assigned to different treatments replicated in two sites within two areas selected at random along the study dune system at the beginning of the experiment (May 2010). (DOC) [file pone.0047561.s001.doc]

**Table S1**

**PERMANOVA on Euclidean distances of plants assigned to different treatments replicated in two sites within two areas selected at random along the study dune system at the beginning of the experiment (May 2010).**

| **Source** | **d.f.** | **SS** | **MS** | **Pseudo-*F*** | ***P*** |
| --- | --- | --- | --- | --- | --- |
| Area = A | 1 | 4.98 | 4.99 | 2.66 | 0.123 |
| Treatment = T | 7 | 29.7 | 4.24 | 1.05 | 0.387 |
| Site(A) = S(A) | 2 | 3.75 | 1.87 | 0.47 | 0.871 |
| A x T | 7 | 23.11 | 3.30^a^ |  |  |
| S(A) x T | 14 | 57.57 | 4.12^a^ |  |  |
| Residual | 64 | 260.9 | 4.08^a^ |  |  |
| Pooling^a^ | 85 | 341.57 | 4.02 |  |  |

Variables of interest were number of shoots, rhizome length, number of branches and maximum shoot height. ^a^ Denotes post-hoc pooling, *P* > 0.25; new *F*-values are given for those tested against the pooled term. Each test was based on 9999 permutations of residuals under the reduced model.
