# Supplementary material for: Global Change and Response of Coastal Dune Plants to the Combined Effects of Increased Sand Accretion (Burial) and Nutrient Availability
Source: PLoS One. 2012 Oct 15;7(10):e47561. doi: 10.1371/journal.pone.0047561 (PMC3471884; doi:10.1371/journal.pone.0047561)
Supplement: Table S2 — PERMANOVA on Euclidean distances of plants subjected to different treatments replicated in two sites within two areas selected at random along the study dune system at the end of the experiment (August 2010). (DOC) [file pone.0047561.s002.doc]

**Table S2**

**PERMANOVA on Euclidean distances of plants subjected to different treatments replicated in two sites within two areas selected at random along the study dune system at the end of the experiment (August 2010).**

| **Source** | **d.f.** | **SS** | **MS** | **Pseudo-*F*** | ***P*** |
| --- | --- | --- | --- | --- | --- |
| Area = A | 1 | 5.12 | 5.12 | 0.51 | 0.756 |
| Burial = B | 3 | 59.06 | 19.69 | 2.13 | 0.083 |
| Nutrient = N | 1 | 60.26 | 60.26 | 2.15 | 0.208 |
| Site(Area) = S(A) | 2 | 19.98 | 9.99 | 1.32 | 0.211 |
| A x B | 3 | 27.74 | 9.25 | 1.22 | 0.249 |
| A x N | 1 | 28.01 | 28.01 | 3.69 | 0.005 |
| B x N | 3 | 40.34 | 13.45 | 1.77 | 0.041 |
| B x S(A) | 6 | 37 | 6.17^a^ |  |  |
| N x S(A) | 2 | 14.9 | 7.47^a^ |  |  |
| A x B x N | 3 | 16.92 | 5.64^a^ |  |  |
| B x N x S(A) | 6 | 39.95 | 6.65^a^ |  |  |
| Residual | 64 | 505.67 | 7.90^a^ |  |  |
| Pooling^a^ | 81 | 614.48 | 7.58 |  |  |

Variables of interest were maximum shoot height, mean shoot internode length, number of shoot internodes, number of shoots, rhizome length, number of branches, and biomass of shoots, rhizome and roots.  ^a^ Denotes post-hoc pooling, *P* > 0.25; new *F*-values are given for those tested against the pooled term. Each test was based on 9999 permutations of residuals under the reduced model.
