# Supplementary material for: Global Change and Response of Coastal Dune Plants to the Combined Effects of Increased Sand Accretion (Burial) and Nutrient Availability
Source: PLoS One. 2012 Oct 15;7(10):e47561. doi: 10.1371/journal.pone.0047561 (PMC3471884; doi:10.1371/journal.pone.0047561)
Supplement: Table S3 — Results of a posteriori pair-wise comparisons for the significant interaction terms, burial × nutrient and area × nutrient, detected by PERMANOVA (Table S2). (DOC) [file pone.0047561.s003.doc]

**Table S3**

**Results of *a posteriori* pair-wise comparisons for the significant interaction terms, burial x nutrient and area x nutrient, detected by PERMANOVA (Table S2).**

| **Burial x Nutrient** | **Contrast** | ***t*** | ***P*(MC)** |
| --- | --- | --- | --- |
| No burial | N-, N+ | 5.49 | 0.002 |
| Artifact control | N-, N+ | 1.69 | 0.463 |
| Partial burial | N-, N+ | 1.23 | 0.352 |
| Complete burial | N-, N+ | 0.59 | 0.813 |
| No nutrient added | NB, AC | 0.7 | 0.738 |
|  | NB, PB | 2.12 | 0.083 |
|  | NB, CB | 2.38 | 0.097 |
|  | AC, PB | 1.33 | 0.306 |
|  | AC, CB | 2.47 | 0.068 |
|  | PB, CB | 1.83 | 0.162 |
| Nutrient added | NB, AC | 0.88 | 0.596 |
|  | NB, PB | 0.94 | 0.544 |
|  | NB, CB | 1.65 | 0.176 |
|  | AC, PB | 1.18 | 0.362 |
|  | AC, CB | 1.85 | 0.127 |
|  | PB, CB | 2.36 | 0.036 |
|  |  |  |  |
| **Area x Nutrient** | **Contrast** | ***t*** | ***P*(MC)** |
| Area 1 | N-, N+ | 2.56 | 0.047 |
| Area 2 | N-, N+ | 2.01 | 0.079 |
| No nutrient added | A1, A2 | 1.67 | 0.086 |
| Nutrient added | A1, A2 | 1.03 | 0.427 |

Levels of nutrient (N- = no nutrient added, N+ = nutrient added) and burial (NB = no burial, AC = artifact control, PB = partial burial, CB = complete burial), and nutrient and area (A1, A2 = area 1 or 2) are compared. *t*-test probabilities are uncorrected results generated by PERMANOVA on paired groups. Each test was based on 9999 permutations of residuals under the reduced model. Monte-Carlo probability values, *P*(MC), were chosen.
